# Independent component analysis reveals new and biologically significant structures in micro array data

**DOI:** 10.1186/1471-2105-7-290

**Published:** 2006-06-08

**Authors:** Attila Frigyesi, Srinivas Veerla, David Lindgren, Mattias Höglund

**Affiliations:** 1 Department of Cardiology, University Hospital, SE-221-85Lund, Sweden; 2 Centre for Mathematical Sciences, Mathematical Statistics, Lund University, SE-223 62 Lund, Sweden; 3 Department of Clinical Genetics, Lund University Hospital, SE-221-85Lund, Sweden

## Abstract

**Background:**

An alternative to standard approaches to uncover biologically meaningful structures in micro array data is to treat the data as a blind source separation (BSS) problem. BSS attempts to separate a mixture of signals into their different sources and refers to the problem of recovering signals from several observed linear mixtures. In the context of micro array data, "sources" may correspond to specific cellular responses or to co-regulated genes.

**Results:**

We applied independent component analysis (ICA) to three different microarray data sets; two tumor data sets and one time series experiment. To obtain reliable components we used iterated ICA to estimate component centrotypes. We found that many of the low ranking components indeed may show a strong biological coherence and hence be of biological significance. Generally ICA achieved a higher resolution when compared with results based on correlated expression and a larger number of gene clusters with significantly enriched for gene ontology (GO) categories. In addition, components characteristic for molecular subtypes and for tumors with specific chromosomal translocations were identified. ICA also identified more than one gene clusters significant for the same GO categories and hence disclosed a higher level of biological heterogeneity, even within coherent groups of genes.

**Conclusion:**

Although the ICA approach primarily detects hidden variables, these surfaced as highly correlated genes in time series data and in one instance in the tumor data. This further strengthens the biological relevance of latent variables detected by ICA.

## Background

The genome project has greatly increased our knowledge of genome sequences, the genes that they encode, and made it possible to investigate diverse physiological and disease conditions in detail. However, owing to the layered complexity of biological systems, studying one gene or one protein at a time is not a rational approach. The simultaneous analysis of a large number of genes to examine alterations in gene expression i.e., expression profiling, is a more promising approach. The most powerful applications of molecular profiling involve the study of patterns of gene expression alterations across many samples representing sets of cellular responses, phenotypes, or conditions. The simplest way to identify genes of potential interest is to search for those that are consistently either up- or down regulated across similar conditions. To this end, a simple statistical analysis of gene-expression levels will be adequate. However, identifying patterns of gene expression and grouping genes into expression classes might provide much greater insight into biological function and relevance and several statistical techniques have been used for these purposes [1]. Most of these techniques are however analogous in as much as they tend to show the same features of the data represented in different ways e.g., correlation among genes/samples in the appearance of e.g., a hierarchical cluster analysis, K-means clustering, or principal component analysis. However, choosing the appropriate algorithms for analysis is a crucial element of the experimental design and will affect the type of information that is retrieved.

An alternative approach to uncover biologically meaningful structures in data is to treat micro array data as a blind source separation (BSS) problem [2,3]. BSS attempts to separate a mixture of signals into their different sources and refers to the problem of recovering signals from several observed linear mixtures. In the context of micro array data, "sources" may correspond to specific cellular responses or to co-regulated genes. The strength of the BSS model is that only mutual statistical independence between the source signals is assumed and an *a priori *information about, e.g., the characteristics of the source signals or the mixing matrix, is not needed. A frequently used BSS approach is independent component analysis (ICA) using the FastICA algorithm [4]. This algorithm is based on the identification of non-Gaussian components in a sample space under the assumption that Gaussian distributions represent noise. The identification of non-Gaussian, typically super-Gaussian, is biologically relevant in an expression profiling situation as most genes e.g. house keeping genes, are not expected to change at a given physiological/pathological transition, and thus conform to a Gaussian distribution. Only the genes that constitute the physiological/pathological state will change and thus produce super-Gaussian distributions. Liebermeister [5] applied the FastICA algorithm to the yeast cell cycle and B-cell lymphoma data and proposed that the expression profiles were determined by hidden regulatory variables, "expression modes", identified as ICA components. Lee and Batzoglou [6] evaluated the efficiency of different variants of ICA procedures, including both linear and non-linear alternatives. The results obtained were compared to other commonly used clustering algorithms. The evaluation was conducted by comparing the number of biologically significant and coherent gene clusters that was obtained, as determined by gene ontology (GO) term analysis. The authors conclude that ICA outperformed all methods used in the comparison. Martoglio et al. [7] made use of the fact that the expression profiles of the samples ***S ***is a linear mixture of the components ***C ***i.e., in matrix notation ***S ***= ***A ***× ***C***, and used ***A ***to identify components characterizing ovarian tumor subtypes and thus show that ICA may be used for disease (tumor) classification. A similar approach was used by Saidi et al. [8] in the analysis of endometrial cancer. Zhang et al. [9] has shown that ICA may be used for classification of colon and prostate tumors.

In contrast to principal component analysis (PCA), all ICA algorithms face the problem of convergence to local optima, thus slightly different components will be produced when the same data is reanalyzed. In a worst case scenario the algorithm will be trapped in a local optimum. Furthermore, most ICA algorithms return the number of components specified without any indications as to which ones are the more stable. To solve some of these problems Chiappetta et al. [3] constructed consensus components by rerunning the FastICA algorithm with random initializations and by only including components that passed certain criteria of stability in the final analysis. Himberg et al. [10] also introduced re-sampling of ICA components and used estimated centrotypes as representatives of ICA components. In the present investigation we further evaluate ICA as a tool for micro array analysis and particularly focus on the biological counterparts of components. We show that hidden, latent variables identified by ICA may in certain datasets surface as clusters of correlated genes and hence that "expression modes" identified by ICA have distinct biological correlates.

## Results

We used the acute myeloid leukemia (AML) data set described by Bullinger at al. [11]. Cases with a high frequency of missing values were excluded, reporters for identical genes merged, and genes with at least 80% values selected and corrected for missing values by KNN (k-nearest neighbor) imputation. The final data set included 4651 genes and 108 cases. To prevent over-learning the dimension of the data was reduced using PCA so as to maintain 90% of the variance. This reduced the dimension, and hence the maximum number of components, to 60. The FastICA was iterated with 50 randomized initial conditions and each series of iterations repeated 5 times. We used the cluster quality index, I_Q_, to evaluate the resampled components and to obtain support for the most suitable number of components. The I_Q _estimates ordered the components almost according to rank. As no threshold I_Q _value that distinctly identified reliable components could be establishes all 60 components from a run using 50 randomized initial conditions were selected for further analyses.

**Figure 1:**
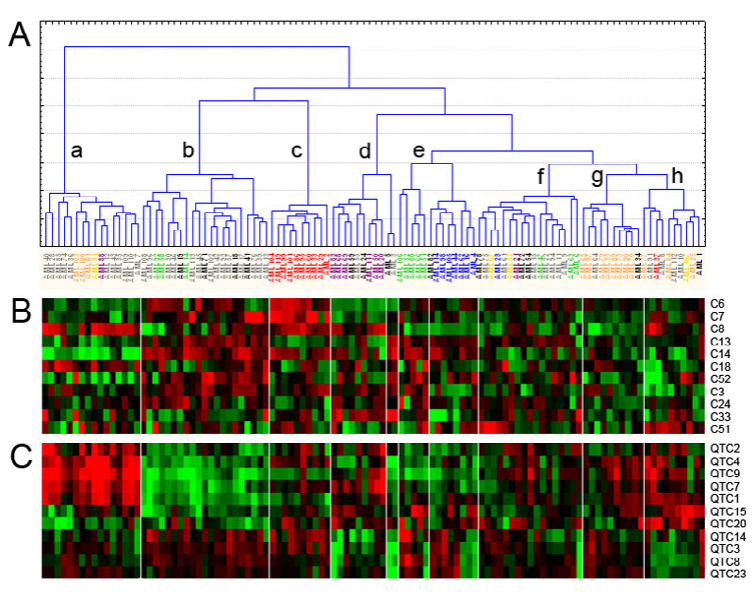
Hierarchical cluster analyses of the AML data set. A) Hierarchical cluster analysis of AML cases using genes assigned to at least one component. Cluster names (a-h) as indicated. Color codes for chromosomal aberrations; grey, normal karyotype; orange, inv(16); yellow, +8 sole; purple, t(9;11); green, del(7q)/-7; black, complex karyotype; red, t(15:17); blue, t(8;21). B) The aligned heat map of ***A ***values for the corresponding components. C) The aligned heat map of the median expression values for the corresponding QTC clusters.

To identify genes associated with specific components, the gene with the highest absolute loading on a given component was retrieved and the remaining data tested to fit a normal distribution using the Lilliefors' test. This was repeated until the remaining data converged to a normal distribution using a predefined p-value of 0.15. The retrieved genes were then considered to be associated with the component. Two of the components, C5 and C27, did not show convergence and was excluded from further analysis. This procedure identified 2271 genes with significant loading on at least one component and hence the original data set was condensed to 36% of the original number of genes. The results from a hierarchical cluster analysis (HCA) using the reduced number of genes is seen in Figure 1a.

The number of genes per component ranged from 8 to 351, with the majority of components having 30–100 gene members (See Additional file 1 for genes in each component). As expected several genes, 50%, were present in more than one component, Table 1. The top 10 genes most frequently participating in components were *CPVL *(13), *DPPA4 *(13), *LOC92235 *(12), *LY86 *(12), *SERPINEG1 *(12), *TACSTD2 *(12), *P2RY5 *(11), *ADM *(10), *C9orf58 *(10), and *GABBR1 *(10). The significant genes for each component were then subjected to GO term analysis using the software EASE. In total 8 components were significant for GO term categories (Table 2). The most significant were "nucleosome assembly" (component 8, C8) and "mitotic cell cycle" (C17) with corrected EASE scores of 4.4 × 10^-16 ^and 2.5 × 10^-29^, respectively. By treating genes with negative and positive loadings on the components separately, six additional components showed significant GO terms, whereas one component (C52) lost significant EASE scores (Table 2). Notably, four different components showed significant EASE scores for the GO category "defense response".

**Figure 2:**
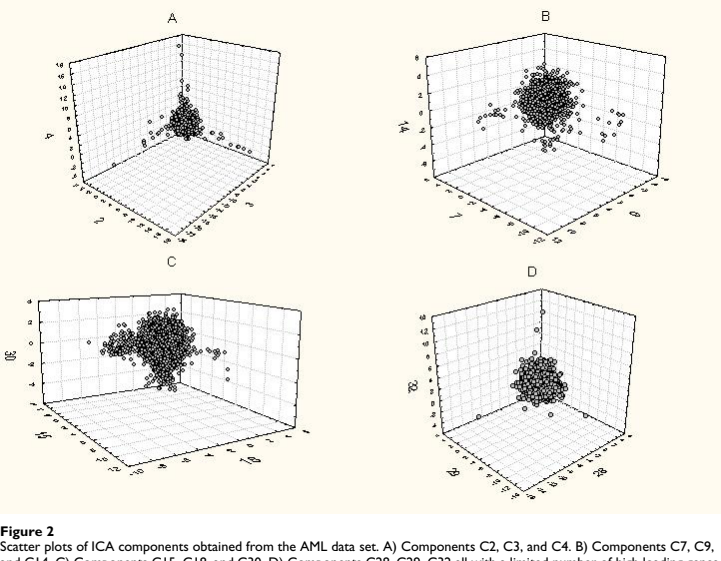
Scatter plots of ICA components obtained from the AML data set. A) Components C2, C3, and C4. B) Components C7, C9, and C14. C) Components C15, C18, and C30. D) Components C28, C29, C32 all with a limited number of high loading genes.

We then inspected components individually by producing scatter plots of the loading values. Several components showed complex structures with groups of genes showing substantial loadings (Figure 2) whereas others showed "simple "structures with the majority of the loadings forming a dense cluster close to the origin and with only a few genes with substantial loadings. This was particularly evident for C1 were the gene with highest loading had a value 15 times the next value. In total 22 components were identified that either showed significant GO term categories or complex patterns of potential biological significance.

Analysis of the component weights (***A***) revealed several components that were either tumor cluster or chromosomal aberration specific (Figure 1b). Component 6 showed particularly low weights in clusters *a *and *d*, of which cluster *d *correspond to the majority of t(9;11) AMLs, but high weights in t(15;17) AMLs. The t(15;17) AMLs also showed height weights for C7. Component 8, associated with "nucleosome assembly", showed particular high weights in tumor clusters *a *and *c*. The four components C3, C24, C33, and C51, all characterized by the GO category "defense response", showed a complex distribution across the tumors and overlapped in their weight profiles.

Gene clusters obtained by ICA were then compared with groups of co-expressed genes as determined by correlation. We used the QT clust algorithm to identify clusters of co-expressed genes. In total 23 clusters were identified and seven of these showed significant enrichment of GO term categories (Table 2). Three of the significant GO categories, "immune response", "extracellular" and "mitotic cell cycle" associated with the QTC clusters were also found among the ICA clusters. The median expression value for each QTC gene cluster was calculated and aligned to the dendogram in Figure 1 (Figure 1c). QTC gene clusters 1, 7, 9, 15, and 20 showed high expression in tumor cluster *a*, and low in *b*. Only a few gene clusters showed tumor type/cluster specific expression. No obvious link between ICA and QTC gene clusters could be observed.

**Figure 3:**
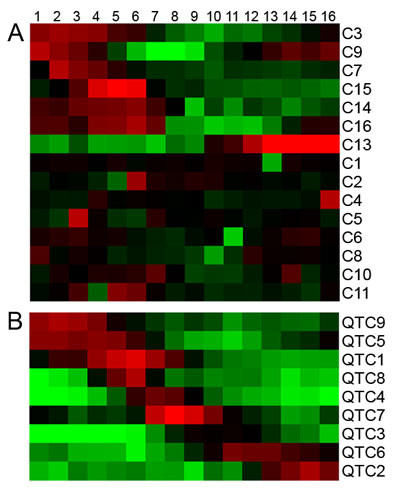
ICA and QTC analyses of the serum induced expression response. A) A heat map of the ***A ***values for the corresponding ICA components. Components C3, C9, C7, C15, C14, C16, and C13 organized according to temporal appearance. B) A heat map of the median expression values for the corresponding QTC clusters. QTC clusters organized according to temporal appearance.

We then analyzed the serum induced gene expression described by Chang et al. [12]. This data set differs from the previous by being of low dimension and by consisting of only 568 genes. Due to the low dimension, 16 time points, sixteen components was derived using 50 randomized initial conditions. ICA reduced the number of genes to 557. All but one component (C12) converged to a normal distribution. The number of genes per component ranged from 5 to 86 and 48% of the genes were present in more than one component (See Additional file 2 for genes in each component). The top ten genes most frequently participating in components included *AREG *(5), *SLC16A6 *(5), *ALDH1A1 *(4), *ANGPTL4 *(4), *BRDT *(4), *C14orf06 *(4), *C4BPB *(4), *CMAH *(4), and *EGR1 *(4). The genes in each component were subjected to GO term analysis and C13 was found to show significant enrichment for GO categories related to the cell cycle, *e.g.*, for "mitotic cell cycle" with a corrected EASE score of 3.4 × 10^-12^. To investigate the influence of each component on the expression profile across the time points the component weights (***A***) were used to construct a heat map, Figure 3A. The distributions of the values of ***A ***revealed single outliers for C1, C2, C4, C5, and C6, whereas seven of the components showed a clear temporal distribution of the ***A ***values. The cell cycle component C13 showed high ***A ***values during the later stages of the serum induced expression.

Groups of co-expressed genes were then identified by the QTC algorithm. Nine clusters of genes were identified and the number of cluster members ranged from 11 to 114. By aligning the median values for each gene cluster with the times points a clear temporal order with regards to peak expression could be seen. The GO analysis revealed two clusters with enriched GO categories, QTC1 with a corrected EASE score of 1.7 × 10^-3 ^for "lipid biosynthesis" and QTC2 with a corrected EASE score of 1.8 × 10^-15 ^for "mitotic cell cycle". Figure 3 indicates both positive and negative correlations between ***A ***values and median values for QTC clusters. A subsequent correlation analysis revealed significant strong positive (r > 0.80) and negative (r < -0.80) correlations between weights for several components and QTC cluster median expression values (Table 3). Hence, in the time series data a link between QTC gene clusters and ICA components genes is seen.

**Figure 4:**
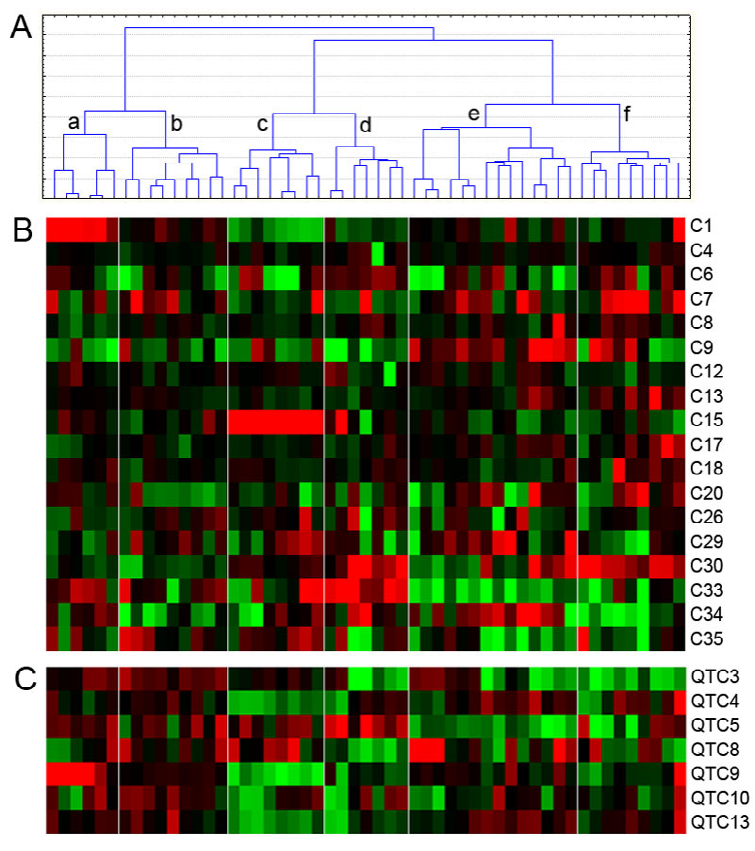
Hierarchical cluster analyses of the HNSCC data set. A) Hierarchical cluster analysis of HNSCC cases using genes assigned to at least one component. Cluster names (a-f) as indicated. B) The aligned heat map of ***A ***values for the corresponding components. C) The aligned heat map of the median expression values for the corresponding QTC clusters.

We then analyzed the head and neck squamous cell carcinoma (HNSCC) expression data described by Chung et al. [11]. The data was downloaded excluding expression profiles obtained from duplicate biopsies. Reporters for identical genes were merged and genes with at least 80% values were selected and corrected for missing values by KNN imputation. This produced a data set comprising 8620 genes and 53 cases. The dimension of the data was then reduced to 35 to maintain 90% of the variance. Iterated FastICA was applied in five runs using 50 randomized initial conditions and the I_Q _indices evaluated. As in the case of the AML dataset no clear distinction between reliable and unreliable components could be established. Consequently the maximum number of components, 35, was retrieved. Our procedure reduced the number of genes from 8620 to 4665 using p-value of 0.01 in the Lilliefors' test for normality. All but one component (C3) converged to a normal distribution. The number of genes per component ranged from 36 to 726 and 2551 genes (55%) were present in more than one component (See Additional file 3 for genes in each component). The top ten genes most frequently participating in components included *UPK1B *(16), *C20orf114 *(14), *CRISP3 *(14), *SERPINB *(14), *GOS2 *(13), *KSP37 *(13), *MMP7 *(13), *PSPHL *(13), and *BCL3 *(12). A HCA performed by using all genes that were present in at least one ICA component produced three major branches of clusters that could be subdivided further into the six sub-clusters *a *to *f *(Figure 4a).

The genes assigned for each component were then subjected to GO term analysis and 17 components were found to be significant for GO term categories (Table 4). The most significant were "immune response" (C6), "extracellular matrix" (C9), "defense response" (C30) and "muscle fiber" (C1) with corrected EASE scores of 5.7 × 10^-32^, 2.3 × 10^-28^, 5.8 10^-17^, and 6.0 × 10^-14^, respectively. By treating genes with negative and positive loadings on the components separately the EASE scores increased and one additional component showed significant GO terms (Table 4). Intriguingly, several of the lower ranking components (C20 to C35) showed considerable EASE scores, *e.g.*, C30, indicating biological significance and six components were significant for the category "extracellular". The component weights (***A***) were used to construct a heat map that was aligned to the dendrogram in Figure 4a. The first component, significant for the GO category "muscle fiber", showed particularly high weights in cluster *a *and low weights in cluster *c *tumors. The tumors displayed specific expression patterns of the "extracellular" components. Component 9 showed predominantly high weights in cluster *e *tumors, C15 in cluster *c *tumors, C30 in cluster *d *and *f *tumors, and C33 in cluster *d *tumors. The remaining two "extracellular" components, C18 and C35, showed less distinct patterns of weight distributions.

We identified 13 clusters of co-expressed genes using the QTC algorithm and the same settings as for the AML cases. Seven of the identified groups of co-expressed genes showed significant enrichment for GO term categories (Table 4). The corrected EASE scores for the top ranking GO categories ranged from 10^-14 ^to 10^-8^. In contrast to the ICA analysis, the QTC produced only one cluster significant for the GO category "extracellular". The median gene cluster expression profiles were used to produce a heat map aligned to the HCA clustered tumors in Figure 4a. Some correlation with tumor subclusters could be seen e.g., QTC3 showed low expression in cluster *f *but relatively high in clusters *a*, *b*, and *c*, and QTC5 showed low expression in cluster *e *but relatively high in clusters *a*, *c*, and *d*. Interestingly, the median expression values for QTC9 ("muscle fiber") showed a strong correlation (r = 0.972) with the weights (***A***) for C1, also significant for the category "muscle fiber".

## Discussion

In the present investigation we have used a blind source separation (BSS) methodology to estimate linear mixtures of statistically impendent sources in micro array data. The fact that of BSS identifies latent variables, or sources, that ultimately produce an overall "profile" is attractive from a biological point of view as these sources may be used as first approximations of expression modules [5,14]. Unlike principal component analysis, most BSS procedures, such as ICA, are based on minimization of an objective function in a large dimensional space; hence most algorithms are related to gradient descent and sensitive to initial conditions. One approach to estimate less unstable components is to iterate the algorithm using different random initializations and to only consider components repeatedly obtained as reliable [3]. A further development of this approach, employed in the present investigation, is to use centrotypes of repeated estimates of the same component [10].

A central issue associated with ICA is the number of components to extract and, the related issue, how to identify the most relevant ones. For the larger datasets, AML and HNSCC, we performed an initial dimension reduction using PCA to the number of dimensions explaining 90% of the variance. There are alternative approaches, however, a too harsh initial dimension reduction may result in loss of biologically relevant components, and a too gentle may leave to many dimensions to be analyzed. Consequently, we choose to be conservative and trimmed the data by excluding only a small (10%) proportion of the variance in. In spite of this the number of dimensions was reduced to 60 (56%) and 35 (66%) in the AML and HNSCC data sets, respectively. A similar dimension reduction was not applied in the Chang data due to the few original number of measurements. Several cluster validity indices have been described [15] and Himberg et al. [10] suggests the cluster quality index, I_q _to be used in connection with iterated FastICA. The I_q _index is a measure of the compactness of the cluster of component estimates and is consequently an estimate of centrotype variance. However, this index was not informative in our hands and as an alternative, we systematically investigated the loading distributions of all extracted components, the distributions of ***A ***values across the samples, and the biological coherence of the formed gene clusters.

Scatter plots produced by combining components revealed a decrease in component information, as determined by scatter structure, with decreasing rank. As a result rank may by itself be used as a selection criterion. However, a closer inspection revealed some high ranking components with one or two very high loading genes *i.e.*, outliers. As we do not know the underlying structure of the data we cannot exclude the significance of such components. However, we believe it is reasonable to assume that changes in cellular states are associated with specific biological processes and that such proceses generally involve large sets of genes. Hence, components with one or two very high loading genes may be hard to interpret in a biological context. The distributions of the ***A ***values gave further information on the possible significance of components. Given that the experimenter has covered the space of investigation with a relevant number of measurements i.e., cases, narrow ***A ***distributions with outliers indicates that the component may identify a very rare condition, or even an artifact, and hence may be of lesser importance. The analysis of the time series data exemplified both the presence of sources with distinct outliers as well as ***A ***distributions hard to fit in to the biological process under study. The time series data describes gene expression changes induced by serum and accordingly involves an *a priori *continuous change from one state to another, given that measurements have been made densely enough. Of the 16 components extracted eight showed either component loading patterns or ***A ***value distributions hard to fit a model of a continuous change in gene expression. Notably, six of these C1, C2, C4, C5, C6, and C8, were among the eight top ranking components with respect to departure from normality as well as with respect to I_q_. All in all only seven components could, at the present level of investigation, be associated with serum induced gene expression, and the majority of these components were low ranking. We conclude that at present, and with the algorithms used, the most reliable way to select components is to manually inspect and evaluate these in light of their component loading and ***A ***distribution as well as by their biological coherence.

To associate genes with specific components Leibermeister [5] selected genes that had loadings on the components larger than four standard deviations. Lee and Batzoglou [6] applied standard deviation thresholds adjusted to the data by evaluating different thresholds using GO-term analysis. In this way the biological coherence of the components were used to determine the gene members. We made use of the assumption that Gaussian distributions represent noise and that FastICA specifically selects non-Gaussian distributions. We reasoned that by removing the genes with the largest loadings in an iterative way and by testing the remaining distribution for normality at each step until convergence was obtained, the most important genes in the ICA component would be extracted. The remaining genes would represent noise signals. In addition, with a specified threshold value for convergence the number of genes associated with a given component is determined by characteristics of the loading distributions only. This approach is similar to the one applied by Lee and Batzoglou [6] in as much as it is tunable, through the p value in the Kolmogorov-Smirnov test, but differs in that fewer genes will be retrieved when the original distribution approaches normality. We encountered a few instances when convergence was not obtained. However, the few non-converging components showed a high proportion of genes with loadings equal to zero, suggesting these components to be extracted by over-learning [16].

I contrast to other gene grouping methods ICA allow genes to be present in more than one gene cluster i.e., component. This feature is attractive as specific genes may participate in different types of cellular responses or signaling/metabolic pathways. We found a large proportion, ~50%, of the genes to participate in more than one component. This proportion is, however, partly dependent on the convergence threshold value, which influences the number of genes assigned to each component. We could not find any patterns or common characteristics of the genes most frequently occurring in components. One could have anticipated that highly linked genes such as *KRAS2 *and *TP53 *that are involved in several signaling pathways would participate in more than one component. On the other hand, genes representing lower hierarchical levels of signaling pathways are frequently shared by more than one pathway and would thus be as likely candidates. Hence, the biological significance of genes present in more than one source needs further investigation.

We used GO term analyses to investigate the biological coherence of the genes associated with each component and 8 of 55 AML and 17 out of 35 HNSCC components showed significant EASE scores. These numbers increased to 15 and 18, respectively, when genes with negative or positive loadings on the components were treated separately. Notably, several low ranking components such as C45, C49, C51, and C52 in the AML data and C30, C33, C34, and C35 in the HNSCC data showed enrichment for GO categories with highly significant EASE scores. This further emphasizes the disparity between significances as determined by component rank and/or the I_q _index and biological significance.

The GO term analysis also revealed that ICA grouped genes at a higher level of resolution than clustering based on correlated expression. Fifteen biologically coherent groups of genes were identified by ICA in the AML data set and 18 in the HNSCC dataset, whereas the corresponding number of coherent clusters formed by correlation was seven in both cases. In addition, six different groups of genes related to the GO category "extracellular" was identified by ICA in HNSCC whereas only one cluster of genes was related to the category "extracellular" after QTC clustering. Similarly, the single cluster of "defense response" detected in the AML set by the QTC algorithm was extended to four clusters using FastICA. This shows that ICA may distinguish between different modifications of the "extracellular" and "defense response" profiles not distinguished by standard approaches. That these differences may be of importance for the behavior of the tumor was shown by the association of the "extracellular" components C9, C15, C30, and C33 with specific molecular subtypes of HNSCC.

Latent variables may, from a biological point of view, appear elusive and merely correspond to algorithmic tools used for describing complex data. It is therefore important to find possible biological correlates to ICA components. A first step in this process has been accomplished by GO term analyses which convincingly have shown that ICA components correspond to groups of biologically coherent genes (Lee and Batzoglou [6], and the present investigation). A further hint to the component nature is the present finding that latent variables (components) may surface as highly correlated genes. This was particularly evident in the times series data in which all components that had been judged as informative by the component loadings and ***A ***distributions, also showed strong correlations with identified QTC gene clusters. In addition, the "muscle fiber" component (C1) in the HNSCC data showed an almost identical behavior, as determined by the ***A ***values, as the QTC cluster 9, also characterized by "muscle fiber" genes. Taken together, these findings further emphasize the biological relevance of latent variables identified by ICA.

## Conclusion

We have shown that independent component analysis may contribute to a deeper understanding of gene expression data. Particularly, ICA resolves expression data at a higher resolution than is achieved by approaches based on correlations alone. In addition, we have further elucidated the biological significance of latent variables identified by ICA. Even though the aim of the present investigation was not to evaluate specific ICA algorithms and procedures, we note that several aspects of the procedure, e.g., indices for reliable components and alternative contrast functions, needs to be evaluated further in the context of micro array data.

## Methods

### Data sets

The AML dataset described by Bullinger et al., [11] was downloaded from the Gene expression Omnibus [19] (accession number GSE425) to contain 6283 genes/reporters. Eleven cases showed a high frequency of missing values (> 1800 missing values) and were excluded from further analyses. Reporters for identical genes were merged and genes with at least 80% values were selected and corrected for missing values by KNN imputation using with K = 12 [17]. The final data set included 4651 genes and 108 cases. The Head and Neck Squamous Cell Carcinoma data set described by Chung et al. [13] was downloaded from the Gene expression Omnibus [19] (accession number GSE686). The data was downloaded excluding expression profiles obtained from duplicate biopsies. Reporters for identical genes were merged and genes with at least 80% values were selected and corrected for missing values by KNN imputation using with K = 12. This produced a data set comprising 8620 genes and 53 cases.

The Time series data described by Chang et al. [12] was downloaded from the Stanford Microarray Database [20]. Reporters for identical genes were merged and genes with at least 80% values were selected and corrected for missing values by KNN imputation using with K = 12 resulting in a dataset of 568 genes and 16 time points. Expression values for t = 0 was obtained by the mean expression values of all experiments designated t = 0.

### Iterated FastICA

We assume that our gene expression (microarray) data is in the form of a matrix ***X ***with rows corresponding to samples and columns corresponding to genes and that it is produced through the linear mixture of independent components ***C***. The relative contribution of each component to the expression profile for a given sample is determined by the coefficients of ***A***, in a matrix form ***X ***= ***A ***× ***C***, of the form (genes × samples) = (samples × component) × (component × genes). The starting point for ICA is the very simple assumption that the components are statistically independent and have non-normal distributions. After estimating the matrix ***A***, we can compute its inverse, ***W***, and obtain the independent components by: ***W ***× ***S***. We performed the ICA using the FastICA-algorithm developed by Hyvärinen A and Oja E. [4]. We used the contrast function g(*u*) = *u*^3 ^(pow3), a variation on the commonly used kurtosis, to identify non-normal components. As the FastICA algorithm relies on random initializations for its maximization of non-normality it will produce slightly different results each time applied. To alleviate this instability we iterated FastICA 50 times (the limitation being set by computation time). The final components were then estimated as the centrotypes of the iterated estimates of each component and evaluated by the I_Q _index as implemented in the Icasso software [10].

### Identification of significant genes in the components

To identify those genes that have significant loadings in each component we assumed that normally distributed loadings represent noise, whereas genes contributing to non-normality were considered significant. We employed an iterative procedure to identify significant genes by removing the gene with the largest absolute loading and then test for normality using Lilliefors' test, a variant of Kolmogorov-Smirnoffs test with unknown mean and variance. Genes were removed one by one until the remaining genes could be considered to be normally distributed. In this manner the number of significant genes in every component depends on the quality of the components, i.e. departure from normality. The p-value in the Lilliefors' test was adjusted for the different data sets in order to retain a suitable number of genes in each component.

### Clustering procedures

We applied two way hierarchical clustering using Euclidean distances and the Ward algorithm for cluster formation to analyze expression data. To find genes with similar expression-profiles we used QT Clust (Quality Cluster algorithm) modified from Heyer et al. [18]. QT Clust proceeds by forming a candidate cluster of the first gene and grouping genes with the highest correlation iteratively in a way that minimizes the cluster diameter d, until no further genes may be added without exceeding a predetermined d-value. This procedure is performed with all genes in the data set as a seed. The largest cluster is then retrieved and the procedure repeated excluding the genes selected for the clusters formed. This makes sure that the largest and most coherent clusters of genes are formed. We used a diameter of 0.3 and a minimal cluster size of 15 members for the AML data and HNSCC data, and a diameter of 0.2 and a minimal cluster size of 10 members for time series data.

### Go-term analyses

We used the EASE software [21] to identify statistically enriched GO term categories. EASE identifies significant enrichment of specific gene ontology (GO) categories in a given list of genes compared to the total list of genes. Step-down Bonferroni multiplicity corrected p-values < 0.05 calculated using EASE statistics were considered significant.

## Authors' contributions

MH conceived the study. AF carried out all the computations. DL and SV performed the bioinformatical analyses and the biological interpretations.

## Supplementary Material

Additional File 1 An excel file with all genes in each ICA component obtained for the AML data. Click here for file

Additional File 2 An excel file with all genes in each ICA component obtained for the time series data. Click here for file

Additional File 3 An excel file with all genes in each ICA component obtained for the HNSCC data. Click here for file

## Figures and Tables

**Table 1:** Genes present in more than one component in the AML data set.^1^

**Gene**	**Number of C^2^**	**Gene**	**Neg load^3^**	**Gene**	**Pos load^4^**
CPVL	13	TACSTD2	8	LY86	9
DPPA4	13	DPPA4	7	P2RY5	8
LOC92235	12	SERPING1	7	GABBR1	8
LY86	12	ADM	7	NOV	8
SERPING1	12	ARHGAP5	7	SCHIP1	8
TACSTD2	12	CLIPR-59	7	CPVL	7
P2RY5	11	EGR1	7	C9orf58	7
ADM	10	GPC4	7	ROBO3	7
C9orf58	10	NBL1	7	ZNF521	7
GABBR1	10	CXCL3	7	C3orf6	7
NOV	10	CPVL	6	DPPA4	6
PRG2	10	LOC92235	6	LOC92235	6
SCHIP1	10	PRG2	6	FCER2	6
SH2D1A	10	C10orf38	6	GOPC	6
AREG	9	HTRA3	6	TPSB2	6
ARHGAP5	9	BHLHB3	6	GUCY1A3	6
C10orf38	9	SPRY2	6	KCNQ1OT1	6
CD200	9	SH2D1A	5	SERPING1	5
CD36	9	AREG	5	SH2D1A	5
CDW52	9	CDW52	5	CD200	5
CLIPR-59	9	KLF12	5	CD36	5
EGR1	9	PPBP	5	MEIS1	5
FCER2	9	SDPR	5	NKX3-1	5
GPC4	9	SCN2A2	5	TRH	5
KLF12	9	SERPINB2	5	AQP1	5
MEIS1	9	COBLL1	5	HOXA4	5
NKX3-1	9	GABRB3	5	KYNU	5
PPBP	9	MMP9	5	ME1	5
ROBO3	9	UGT2B7	5	RSNL2	5
SDPR	9	EDG1	5	S100A12	5
TRH	9	LIN7A	5	CD34	5
ZNF521	9	TNF	5	IL1B	5
AQP1	8	TNFRSF19	5	LGALS2	5
C14orf128	8	DSG2	5	LSP1	5
GOPC	8	EIF5A	5	TNFAIP6	5
HOXA4	8	CD200	4	PALM2-AKAP2	5
HTRA3	8	CD36	4	S100A8	5
IGSF9	8	MEIS1	4	UNC13B	5
IL1R2	8	NKX3-1	4	HBG1	5

^1 ^The top 40 genes in each category are listed.

^2 ^The number of components the gene was assigned to.

^3 ^The number of components in which the gene had a negative load.

^4 ^The number of components in which the gene had a positive load.

**Table 2:** GO analyses of genes assigned to ICA components and to QTC clusters in the AML data set.

	**All genes in component**		**Genes with positive loading**		**Genes with negative loading**	
	
**Gene cluster**	**GO category**	**ES**^1^	**GO category**	**ES**	**GO category**	**ES**
**C3**	extracellular	1.52 × 10^-2^				
	defenes resposne	4.18 × 10^-2^			defenes resposne	4.70 × 10^-3^
**C6**	antigen processing/exogeneous antigen	3.36 × 10^-8^			antigen processing/exogeneous antigen	2.30 × 10^-9^
**C8**	nucleosome assembly	4.39 × 10 ^-16^	nucleosome assembly	1.36 × 10^-17^		
**C13**	inflammatory respons	1.6 × 10^-3^			inflammatory respons	5.12 × 10^-5^
**C14**			inflammatory respons	4.55 × 10^-2^		
**C16**	T-cell receptor complex	2.07 × 10^-5^	T-cell receptor complex	5.4 × 10^-6^		
**C17**	mitotic cell cycle	2.50 × 10^-29^	cell communication	2.76 × 10^-2^	mitotic cell cycle	1.41 × 10^-32^
**C24**					defense respons	7.59 × 10^-6^
**C33**					defense respons	1.57 × 10^-2^
**C42**			extracellular	4.08 × 10^-2^		
**C44**					respons to wounding	1.68 × 10^-2^
**C45**	immune response	6.75 × 10^-7^			immune response	6.44 × 10^-9^
**C49**					Cell-cell adhesion	3.23 × 10^-2^
**C51**					defense respons	2.14 × 10^-2^
**C52**	cell-cell signalling	2.85 × 10^-2^				
						
**QTC1**	immune response	5.5 × 10^-9^				
**QTC2**	extracellular	9.9 × 10^-4^				
**QTC3**	intracellullar	1.6 × 10^-2^				
**QTC4**	mitotic cell cycle	4.3 × 10^-28^				
**QTC8**	nucleus	9.7 × 10^-3^				
**QTC14**	unlocalized	3.4 × 10^-2^				
**QTC23**	organell organization and biogenesis	1.8 × 10^-2^				

^1^ES; Step down Bonferroni corrected Ease scores

**Table 3:** Correlations between component *A *values and the median expression levels of QTC gene clusters^1^

	**QTC9**	**QTC5**	**QTC1**	**QTC8**	**QTC4**	**QTC7**	**QTC3**	**QTC6**	**QTC2**
**C3**	**0.88**	**0.97**	0.49	0.00	-0.45	-0.44	**-0.99**	**-0.66**	-0.20
**C9**	0.43	0.42	-0.45	-0.62	**-0.99**	**-0.81**	-0.47	0.26	0.39
**C7**	**0.88**	**0.79**	0.49	-0.02	-0.39	-0.28	**-0.82**	-0.57	-0.39
**C16**	0.61	**0.90**	**0.63**	0.35	-0.10	-0.35	**-0.86**	**-0.74**	-0.08
**C15**	0.52	**0.65**	**0.87**	**0.76**	0.24	-0.10	-0.62	**-0.80**	-0.45
**C14**	**0.71**	**0.82**	**0.81**	0.44	0.07	-0.09	**-0.81**	**-0.86**	-0.40
**C12**	**-0.73**	**-0.76**	-0.42	0.19	0.20	-0.03	**0.76**	**0.67**	0.25
**C13**	-0.58	-0.41	**-0.87**	-0.46	-0.44	-0.53	0.43	**0.82**	**0.94**
**C1**	0.14	0.10	0.22	0.04	0.06	0.15	-0.16	-0.27	-0.30
**C2**	-0.19	-0.24	0.19	0.34	0.52	0.33	0.23	-0.18	-0.16
**C14**	0.01	0.21	-0.03	0.05	-0.08	-0.08	-0.19	-0.08	0.28
**C15**	0.22	0.06	-0.00	-0.23	-0.08	-0.03	-0.10	-0.11	-0.01
**C6**	0.49	0.56	0.06	-0.20	-0.37	-0.37	-0.50	-0.28	0.28
**C8**	0.35	0.47	-0.11	-0.32	-0.53	-0.52	-0.46	-0.03	0.26
**C10**	0.20	0.49	0.46	0.30	0.17	-0.15	-0.42	-0.44	0.08
**C11**	0.25	0.44	0.54	0.51	0.21	-0.12	-0.41	-0.53	-0.20

^1 ^Significant (p < 0.01) Pearson correlations in bold.

**Table 4:** GO analyses of genes assigned to ICA components and to QTC clusters in the HNSCC data set.

	**All genes in component**		**Genes with positive loading**		**Genes with negative loading**	
	
**Gene cluster**	**GO category**	**ES**^1^	**GO category**	**ES**	**GO category**	**ES**
**C1**	muscle fiber	6.0 10^-14^	muscle fiber	1.1 10^-16^		
**C4**	cell communication	3.8 10^-3^			cell communication	1.0 10^-2^
**C6**	immune response	5.7 10^-32^			defense response	4.1 10^-39^
**C7**	xenobiotic metabolism	9.7 10^-5^	xenobiotic metabolism	9.2 10^-5^		
**C8**	extracellular	2.8 10^-2^				
**C9**	extracellular matrix	2.3 10^-28^	extracellular	2.4 10^-2^	extracellular matrix	6.5 10^-31^
**C12**	extracellular	4.8 10^-2^				
**C13**	extracellular	1.1 10^-2^				
**C15**			extracellular	6.0 10^-4^		
**C17**	membrane	2.5 10^-2^			membrane	4.3 10^-3^
**C18**	extracellular	4.9 10^-2^	extracellular	4.7 10^-3^		
**C20**	morphogenesis	1.5 10^-2^			morphogenesis	1.1 10^-2^
**C26**	extracellular	8.9 10^-3^				
**C29**	extracellular	4.5 10^-5^	extracellular	2.9 10^-5^		
**C30**	defense response	5.8 10^-17^	extracellular	3.4 10^-2^	defense response	5.8 10^-17^
**C33**	ectoderm development	5.7 10^-6^			ectoderm development	1.74 10^-6^
**C34**	response to biotic stimulus	3.6 10^-3^			response to biotic stimulus	2.83 10^-2^
**C35**	extracellular	3.3 10^-5^	extracellular	1.9 10^-3^	epidermal differentiation	2.83 10^-3^
						
**QTC3**	defense respons	5 × 10^-7^				
**QTC4**	ribonucleoprotein complex/ribosome	2.5 × 10^-8^/5.1 × 10^-6^				
**QTC5**	extracellular	1.8 × 10^-9^				
**QTC8**	defense respons/immune respons	1.1 × 10^-14^/3.9 × 10^-14^				
**QTC9**	muscle fiber	7.5 × 10^-21^				
**QTC10**	mitotic cell cycle	9.6 × 10^-8^				
**QTC13**	mitochondrion	4.7 × 10^-8^				

^1^ES; Step down Bonferroni corrected Ease scores
